# Assessing the Population Structure and Invasion Risk in Suitable Areas of the Rice Pest *Leptocorisa acuta* (Hemiptera: Alydidae)

**DOI:** 10.3390/insects16090949

**Published:** 2025-09-10

**Authors:** Xue Dong, Xiuxiu Zhu, Zechen Tang, Wenbo Yi, Wenjun Bu

**Affiliations:** 1 School of Life Science, Shanxi Normal University, Taiyuan 030031, China; dongxuer0123@163.com; 2 Institute of Entomology, College of Life Sciences, Nankai University, Tianjin 300071, China; 3 Department of Biology, Xinzhou Normal University, Xinzhou 034000, China

**Keywords:** rice seed bug, population genetics, potential distribution, rice production regions, single nucleotide polymorphisms

## Abstract

Rice (*Oryza sativa*) provides a major food resource worldwide, playing an important role in the global economy. *Leptocorisa acuta* (Hemiptera: Alydidae), commonly known as the rice seed bug, is a major pest of paddy crops in many rice-growing regions and it is considered a potential invasive pest in the United States. Here, we investigated the genetic structure and demographic history of 18 populations sampled from China and southeast Asia using double-digest restriction site-associated DNA sequencing (ddRAD-seq). Then, we performed niche modeling based on occurrence records under current and future climate scenarios. Our analyses suggested that the lack of genetic structure among populations of *L. acuta* is related to recent diversification, strong flight, and dispersal capability, leading to a high level of gene flow. The demographic history was not strongly affected by the last glacial maximum. Ecological niche modeling predicts that future suitable areas will expand in Asia and America, relative to the current conditions. The ecological niche results demonstrated that *L. acuta* is a potentially invasive pest to the United States (mainly Florida and nearby areas) under current and future scenarios. Moreover, the moderately and highly suitable areas will increase in America (primarily located in North America, namely Florida and nearby areas, and Mexico), Central American and Caribbean countries, and some regions of South America. Some South American countries have extensive rice crops and broadly suitable habitats that may indicate a higher invasion risk. Through population genetics, our study supports the strong dispersal capacity of this insect pest and calls for vigilance against its invasion in some countries in the Americas.

## Simple Summary

*Leptocorisa acuta* is a serious pest that damages rice crops in many parts of Asia. Because rice is a key food source for much of the world, understanding the biology and spread of this insect is important. In this study, we used genetic data from 18 populations across China and Southeast Asia to assess the population structure and dispersal ability of *L. acuta*. We found that this pest shows little genetic difference across regions, suggesting it can move and spread easily. We also used ecological modeling to predict where it might spread in the future. Our results show that the pest is a potentially invasive pest in the Americas, including parts of North, Central, and South America. This highlights the need for early monitoring and prevention efforts in regions where rice is widely grown and where the environment is suitable for the pest to establish.

## 1. Introduction

Rice (*Oryza sativa*) is the world’s most important food crop and is cultivated in more than 100 countries (accessed on 10 May 2023, http://www.fao.org/faostat/en/#data), mainly in monsoon Asia, and is distributed across a wide latitudinal range, spanning the tropical and temperate zones [1]. Rice seed bugs are one of the few pests that feed directly on developing rice spikelets in the field [2,3]. *Leptocorisa acuta* (Thunberg, 1783) (Hemiptera: Alydidae) is the most widely distributed species of rice seed bugs in Asia and Oceania [4,5]. *L. acuta* can be found on many crop plants of the family Poaceae, especially rice, and it is an economically important rice pest in rice-producing countries [6,7,8,9]. *L. acuta* is very common in India (known as the Gandhi bug), Sarawak, and New Guinea, and is present throughout the year [4]. *L. acuta* feeds on rice, leading to losses caused by empty spikelets, small and misshapen kernels, and pecky kernels with unsavory black spots [10,11]. In addition, *L. acuta* is a potentially invasive pest in the United States because of its widespread distribution in many rice-producing countries (accessed on 12 October 2022, https://edis.ifas.ufl.edu/publication/IN1067).

Most (97%) of the recorded host plants fed upon by nymphs or adults were grasses (Poaceae), and the remaining were sedges (Cyperaceae) [12]. These hosts are especially vulnerable to *L. acuta* just after the adults emerge from dormancy and during the period before rice flowering. Rice was more attractive than wild grasses to *L. acuta* [13], and *L. acuta* laid more eggs on rice than on other hosts [14]. After rice has been harvested, adults must find alternative food sources until a second rice crop begins to flower.

Nymphs and adults feed by inserting their needlelike mouthparts into new leaves, stems, and developing grains, which causes the most economically important damage to rice crops [15]. *Leptocorisa acuta* is known to transmit bacteria and fungi, which are the cause of bacterial leaf blight and sheath rot disease [16,17,18]. Sheath rot disease harms the panicles of rice plants and causes underdeveloped or damaged rice grains. In severe cases, the infected plant may not produce rice grains [16]. Currently, the application of chemical insecticides is the primary strategy to control *L. acuta* [19].

There can be up to five generations of *L. acuta* under favorable conditions [18]. The duration of five nymph instars is approximately 21 d on rice [12,18] and they are most abundant under conditions of 26.7–27.8 °C and approximately 80% relative humidity [20]. In addition, *L. acuta* can disperse long distances from grasslands during the off-season to seasonal rice fields suggests the possibility that it has a high flight ability [21]. At present, there are many studies on its harm and control measures, but no molecular genetic markers have been used to study its genetic structure and phylogeographic pattern, and the invasion potential of *L. acuta* are less understood.

Here, we aimed to characterize the genetic structure, demographic history, and invasion potential of *L. acuta*. We sampled *L. acuta* specimens across a large geographical range, and we used nuclear single nucleotide polymorphism (SNP) markers generated by double-digest restriction site-associated DNA sequencing (ddRAD-seq) to gain insights into the current population genetic structure of *L. acuta* and its historical demography. In addition, we used known occurrences for ecological niche modeling to predict species distribution and infer the risk of invasion into the Americas. Considering the importance of this crop as a worldwide food, our work contributes to understanding the evolutionary history and invasion potential of this pest from population genetics and helps to establish control measures in strategic locations.

## 2. Materials and Methods

### 2.1. Sample Collection and DNA Extraction

**Figure 1:**
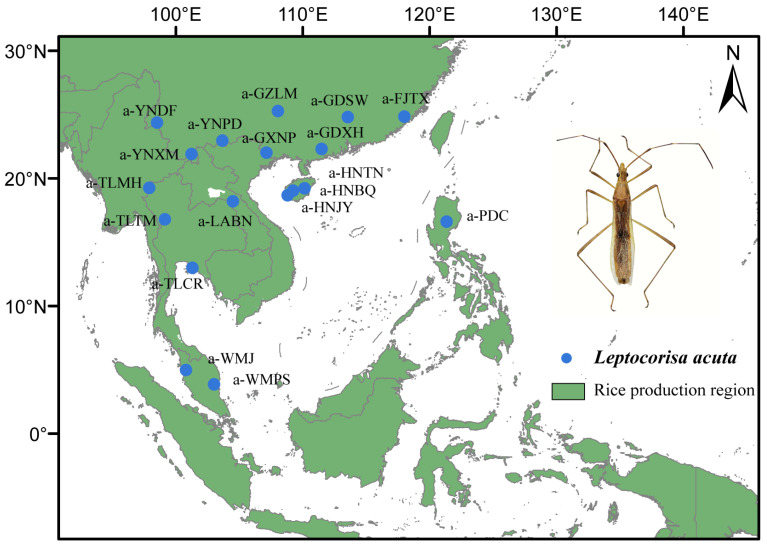
Maps of sampling locations of *Leptocorisa acuta* used in our study.

A total of 83 adult *L. acuta* were collected from 18 localities (both males and females were included in the sampling) where rice is widely cultivated and affected by the pest (Figure 1; Table 1). All samples were preserved in 100% ethanol in the wild and stored at −20 °C in a freezer. Genomic DNA was obtained from each insect using a Universal Genomic DNA Kit (CWBIO, China) according to the manufacturer’s protocol. The DNA and voucher specimens were deposited at the College of Life Sciences at Nankai University, Tianjin, China (NKU). In addition, rice (*Oryza sativa*) cropland maps were generated from the IRRI Dataverse (accessed on 1 June 2023, https://dataverse.harvard.edu/dataset.xhtml?persistentId=doi:10.7910/DVN/JE6R2R) and [22].

### 2.2. ddRAD Sequencing and Data Processing

ddRAD-seq library was prepared to obtain nuclear SNPs following Peterson’s protocol [23]. Briefly, 200–500 ng of DNA was digested using the EcoRI and MspI restriction enzymes (New England Biolabs). The digested DNA fragments were ligated with a unique 5 bp barcode and Illumina adapter. Then, the ligated DNA samples with the same index were pooled and size-selected (200–600 bp) on a Pippin Prep size selector (Sage Science, Beverly, MA, USA) followed by PCR amplification with 8–10 cycles. Amplified DNA fragments from each sample were purified using AMPure XP magnetic beads (Beckman Coulter Inc., Indianapolis, IN, USA) and quantified with an Agilent 2100 bioanalyzer (Agilent Technologies, Santa Clara, CA, USA). The library was sequenced on the Illumina NovaSeq 6000 platform to generate 150 bp paired-end reads.

We used an ipyrad v. 0.9.42 pipeline [24] to demultiplex and assign reads to individuals based on barcodes and then performed subsequent analysis. For the ipyrad parameters, the minimum read depth was set to 6 for calling consensus sequences, and a clustering threshold of 85% was applied for de novo assembly. A final dataset (SNP_80) was generated, with a maximum of 20% missing data per locus allowed across individuals. A USNP_80 dataset was also output to avoid linkage within the same locus for downstream analysis. The sequencing data were deposited in the GenBank database (BioProject accession number: PRJNA1026486).

### 2.3. Population Genetics Analyses

The genetic diversity and population genetic structure were measured based on the SNP_80 dataset (containing 174,797 SNPs). The observed heterozygosity (HO), expected heterozygosity (HE), and nucleotide diversity (π) were calculated in Arlequin 3.5 [25]. Pairwise genetic differentiation statistic (FST) values were estimated in the hierfstat package [26] and corresponding estimate of the gene flow (Nm) is approximated by (1/F_ST_−1)/4 [27,28]. Phylogenetic relationship was performed using the Neighbor-Net method, implemented in SplitsTree v4.14.5 [29].

We used the Bayesian clustering method STRUCTURE v.2.3.4 [30] to assess the level of population structure. Ten replicates were conducted for each K value from 1 to 10 and each run was performed for 500,000 iterations following 100,000 iterations as burn-in. We used STRUCTURE HARVESTER [31] to assess the most likely value of K using the delta K method [32]. Isolation by distance (IBD) was performed by examining the correlation between genetic distance and geographical distance using the Mantel test, implemented in the R package Adegenet 2.1.3 [33] with 10,000 permutations. In addition, we used the Stairway plot method [34], which is reliable for speculating the demographic history over long periods, to investigate the population demographic history of *L. acuta* utilizing a one-dimensional unfolded site frequency spectrum (SFS) generated by the easySFS script (accessed on 15 August 2022, https://github.com/isaacovercast/easySFS).

### 2.4. Ecological Niche Modeling

The maximum entropy algorithm implemented in MaxEnt software 3.4.1 [35] was selected to predict the suitability and evaluate the impacts of environmental factors on the distribution of *L. acuta*. MaxEnt makes inferences by comparing presence points with background points (for which the presence is not known) and has been widely employed because of its small sample size requirements and superior predictive performance [35,36,37]. We downloaded 19 bioclimatic variables from the WorldClim database (http://www.worldclim.org) accessed on 27 September 2023 and gathered 125 occurrences from our fieldwork and the Global Biodiversity Information Facility (GBIF) database (https://www.gbif.org/) and accessed on 10 May 2023. After spatial thinning, 83 occurrence records were left within a 20 km radius. The relative contribution and importance of each variable was evaluated using the Jackknife method by comparing the regularized training gain. To identify the optimal model parameters, five feature classes (FCs: linear (L); linear quadratic (LQ); hinge (H); linear quadratic hinge (LQH) and linear quadratic hinge product (LQHP)) were tested with regularization multiplier (RM) ranging from 0.5 to 4.0 in increments of 0.5 in the Wallace package [38]. Then, to eliminate multi-collinearity and improve the model performance before modeling, the Pearson correlation coefficient was used to assess the correlations among bioclimatic variables, in which |r| ≥ 0.80 was considered to indicate highly correlated variables. Model generation was iterated 15 times, and 75% of the data were randomly selected as a training set with 25% as the testing set. Model performance was evaluated by the receiver operating characteristic (ROC) curve and the value of the area under the ROC curve (AUC) [23]. AUC values range from 0 to 1, with values greater than 0.5 indicating a better performance than employing random selection. A threshold of the maximum training sensitivity plus specificity (MTSPS) was chosen to define the suitable and unsuitable regions of *L. acuta* [39,40].

To generate the potential distribution of *L. acuta* under future climate conditions, we projected the models into the 2050s (average for 2041–2060) and 2070s (average for 2061–2080) in scenarios of low (SSP 126) and high (SSP 585) greenhouse gas emission, available from the WorldClim database. To improve reliability, all future predictions were calculated based on three different global circulation models (GCMs) from Coupled Model Intercomparison Project Phase 6 (CMIP6), including the Canadian Earth System Model version 5 (CanESM5), the Institute Pierre-Simon Laplace coupled model version 6 (IPSLCM6A-LR), and the Max Planck Institute for Meteorology Earth System Model (MPI-ESM1-2-LR), each of which represents different climate sensitivities to future climate change projections [41].

## 3. Results

### 3.1. Nuclear Genetic Diversity and the Lack of Population Structure

**Figure 2:**
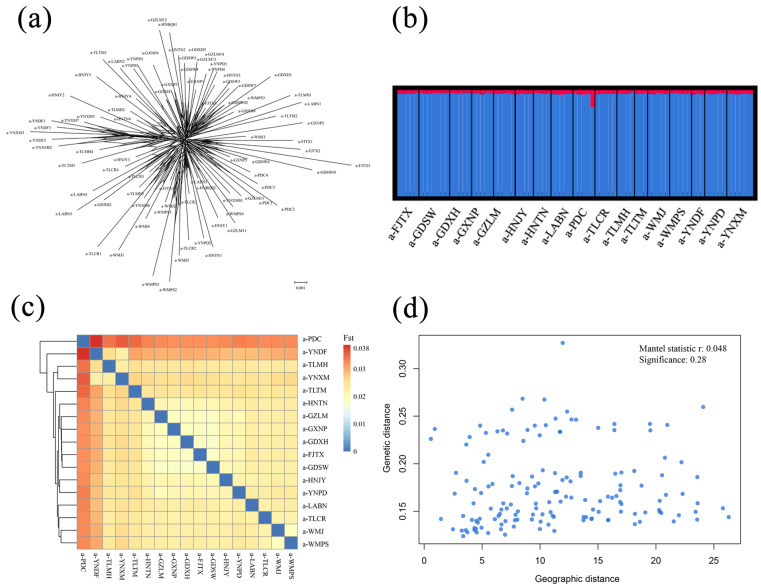
Population genetics analysis results based on double-digest restriction site-associated DNA data of *Leptocorisa acuta* in Asia. (**a**) Phylogenetic topology based on 174,797 SNPs by the neighbor-net method does not show population subdivision. (**b**) Bayesian genetic cluster inferred by Structure (K = 2). Each individual is represented by a vertical bar. (**c**) Pairwise F_ST_ values among populations estimated from SNPs. (**d**) The Mantel test of isolation by distance (IBD) showed the pairwise correlation between genetic and geographic distance among populations of *L. acuta*.

We obtained a total of 615,464,758 reads, with an average of 7,415,238 demultiplexed reads per specimen after removing low-quality reads (Table S1). Genetic diversity was calculated for populations containing more than 3 samples. The populations showed similar levels of nucleotide diversity (π), ranging from 0.0208 to 0.0241 (Table 1). The average expected and observed heterozygosity per locus were calculated (HE ranged from 0.2521 to 0.4138, and Ho ranged from 0.2165 to 0.3660) (Table 1). The Neighbor-Net tree did not show significant population subgroups based on the ddRAD dataset (Figure 2a). The pairwise Fst showed little genetic differentiation among populations (ranging from 0.0183 to 0.0387; Figure 2c; Table S2). And the level of gene flow exceeds the threshold (Nm > 1), which also indicates gene flow between these populations (Figure S1, Nm ranged from 6.2037 to 13.4270), at which the rates of gene flow could offset population differentiation [29]. For population genetic structure, the Bayesian cluster analysis showed the best K = 2 (Figure S2), and all geographic populations contained similar genetic components (Figure 2b). Our IBD test indicated no significant correlations between genetic differentiation (F_ST_)/(1 − F_ST_) and geographical distances (r = 0.048, *p* = 0.28) among populations (Figure 2d). The stairway plot analysis suggested that the effective population size (Ne) of *L. acuta* increased rapidly during the last glacial maximum (LGM, ~25–19 kya) [42] (Figure S3).

### 3.2. The Current and Future Potentially Suitable Areas for L. acuta

**Figure 3:**
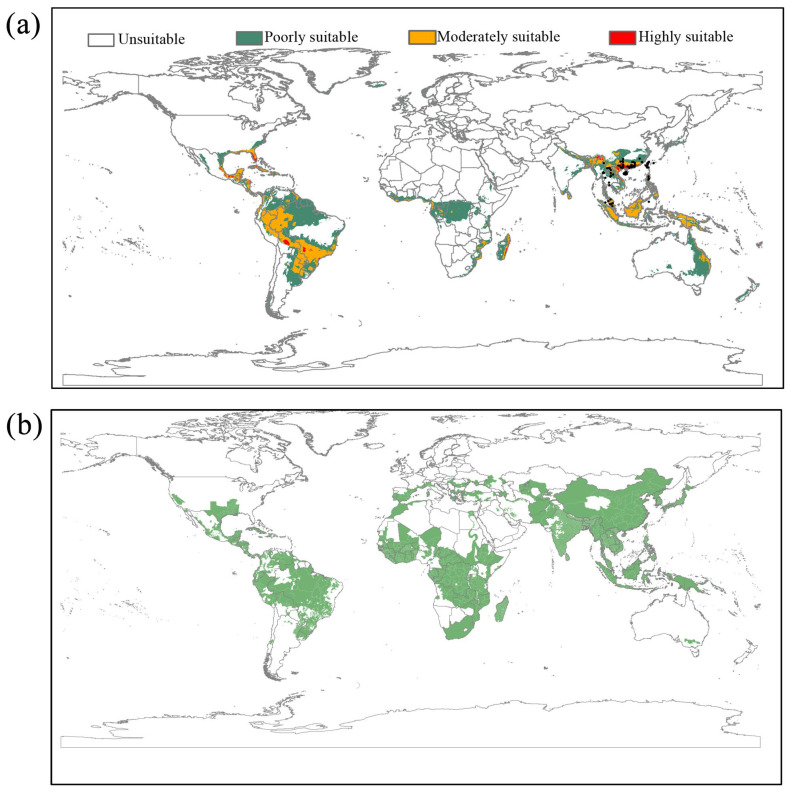
(**a**) Modeled habitat suitability of *Leptocorisa acuta* by the MaxEnt model under current environmental conditions. The black points in the figure represent the occurrence used for modelling. (**b**) The maps of current worldwide rice (*Oryza sativa*) distribution and rice-producing regions covered in RiceAtlas.

According to Pearson’s correlation coefficient and contributions of variable (Table S3), seven environmental variables (BIO2, BIO4, BIO5, BIO8, BIO12, BIO14, and BIO18) were selected for the modeling. The response curves of environmental curves are shown in Figure S4. The parameter combination of FC = H and RM = 4 was chosen to calibrate the habitat suitability model in MaxEnt. A model with an AUC > 0.8 indicates good model performance [43]. The logistic output generated by MaxEnt can be interpreted as an estimate of the relative probability of species distribution in geographical space, with values that vary from 0 (lowest probability) to 1 (highest probability) [36]. The map with current habitat suitability scores for *L. acuta* at the global scale is shown in Figure 3. According to the threshold of MTSPS (0.198), the current distribution model of *L. acuta* indicates suitable areas in North America (east coast of the United States, the coastal regions of Mexico), Central American and Caribbean countries, broad regions of South America, and parts of Africa (coastal and central regions) (Figure 3a). Although *L. acuta* has not yet been reported anywhere other than Asia and the Oceanic countries, the niche model indicates favorable climatic conditions in these locations.

**Figure 4:**
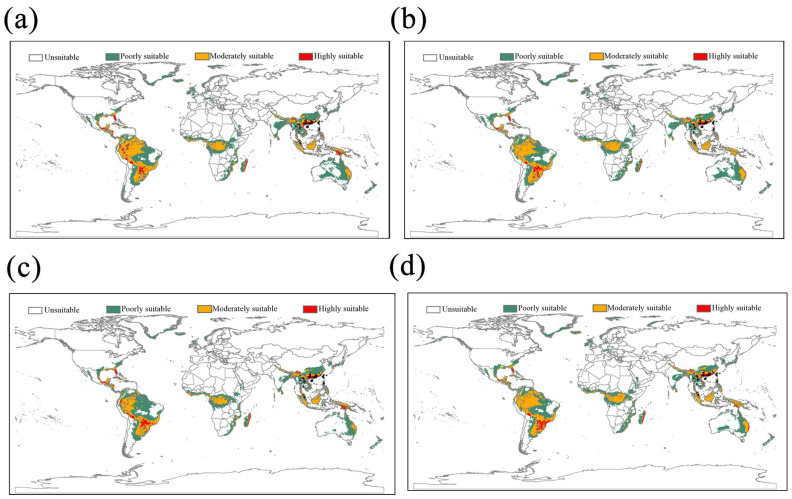
Modeled habitat suitability of *Leptocorisa acuta* under future climate conditions for low Shared Socio-economic Pathways (SSP 126) and high (SSP 585) scenarios in the 2050s (**a**,**b**) and in the 2070s (**c**,**d**) with 83 occurrence records (black points).

For future ecological modeling, MaxEnt analyses were accurate for both SSP scenarios (mean AUC of 0.833 [±0.08]). The suitable areas for *L. acuta* will expand northward in Asia, and the predicted suitable areas in America will also expand under the two climate change scenarios (Figure 4). Moreover, the areas with moderate and high suitability will increase in the Americas, primarily located in the United States (mainly Florida and nearby areas), Central American and Caribbean countries, and wide regions of South America (Figure 4). Rice crops are grown in many countries around the world, and are widely distributed in Asia, Europe, Central and South America, Africa, and Oceania (Figure 3b). Under current and future climate conditions, the suitable habitats of *L. acuta* overlap considerably with areas of rice cultivation.

## 4. Discussion

### 4.1. Genetic Diversity and Population Genetic Structure

Rice domestication had an enormous impact on the people of East, Southeast, and South Asia [44]. Accurate analysis of the genetic structure of a rice pest is of great importance for understanding the pest’s evolutionary history and implementing pest control strategies. To our knowledge, our study is the first to use a genome-wide SNPs to resolve the genetic relationships among populations of *L. acuta* in China and Southeast Asia, the major rice-producing countries. Studies have shown that analyzing thousands of markers provides a fine-scale resolution in the measurement of genetic differentiation among populations [45,46,47].

We conducted genetic diversity statistics to find the variation trend of genetic diversity in the populations. Recently colonized populations may possess decreased genetic diversity, heterozygosity and genetic differentiation [48,49,50,51]. However, no decreased trend among statistics was observed in our analyses (Table 1). Neighbor-net tree, Structure and FST analyses based on the nuclear SNPs datasets revealed low genetic differentiation, no obvious population subgroups and homogeneous genetic components within species may be due to frequent interpopulation gene flow (Figure 2). Furthermore, Nm estimates indicated that high levels of gene flow among populations in China and Southeast Asia were insufficient to prevent population differentiation under migration–drift equilibrium. One of our most surprising results is the finding that the distant oceanic islands of the Philippines have homogeneous genetic component with mainland China (Figure 2b). Most island populations are monophyletic and early-divergent populations with deep genetic structures [52]. However, no subgroups could occur in species with very recent diversification and long-term gene flow between populations across this region, though we have indeed found more apparent genetic differentiation between the Philippines islands (the population a-PDC) and other populations than other combinations of *L. acuta* (Figure 2c). Despite the fact that the collected samples span a period of 10 years, the genetic diversity of the population did not show an association with earlier or later sampling times, indicating this time span is relatively short in the evolutionary history of species and is not enough to have an impact on genetic diversity.

*L. acuta* has a high flight ability and can disperse long distances to find rice in flower [14,53]. Migration usually increases gene flow and reduces genetic differentiation among populations [54,55,56,57]. The presence of alternative hosts within the dispersal distance of adults sustained high adult populations between rice crops [53]. Strong dispersal ability, extensive host use, and large effective population size also affect the lack of population genetic structure. In addition, the population expansion of *L. acuta* was not strongly affected by the LGM (Figure S3), which is consistent with studies of multiple species in East Asia [58,59,60]. In East Asia, many species grew steadily throughout the LGM, as environmental changes appeared to be moderate during the climate oscillations [61].

### 4.2. Potentially Suitable Habitat and Invasion Areas

The availability of shared information systems around the world makes it easier to obtain information on the geographical distribution of several organisms [62]. For those species that have historically attracted attention, there are many experts in the world, so the occurrence and distribution data shared in the databases are reliable. In our study, we integrated occurrence records to perform global niche modeling of *L. acuta* based on bioclimatic variables. In Asia, the predicted currently suitable habitat covers a vast geographic range, including most rice cultivation regions, showing identical or larger distribution scales than those in taxonomic studies [4,53,63]. The modeling results showed that there are potentially suitable regions in North America (notably the east coast of the United States and the coastal regions of Mexico), Central American and Caribbean countries, and wide regions of South America and Africa (coastal and central regions) (Figure 3a). The models show that suitable habitats will expand for projections of climate change scenarios by the 2050s and 2070s (Figure 4, Figure S5). In particular, the moderately and highly suitable areas will increase in America (Figure 4). The ecological niche results demonstrated that *L. acuta* is a potentially invasive pest to the United States (mainly Florida and nearby areas). Due to the large acreage of rice grown in America (Figure 4b), we call for vigilance against the invasion of this pest. The results also suggest that global warming may affect the distributions of insects depending on their ecological needs.

### 4.3. Implications for Management

Rice is one of the most important foods in the world, providing a major source of energy for more than half of the world’s population and playing a substantial role in the global economy [64]. *L. acuta* has attracted wide attention for its reported high economic yield losses in many countries [17,65,66]. Our study revealed the phylogeography and invasion risk of *L. acuta* by population genetics and ecological modeling approaches. Their high dispersal ability, wide host use, and large population size make *L. acuta* a difficult pest to fully control. *L. acuta* was more abundant in rice-growing areas surrounded by alternative grass hosts for several generations before rice flowering [13]. Thus, controlling the surrounding weeds will have some effect on reducing this pest. In addition, we suggest that attention should be given to preventing the invasion of *L. acuta* due to the observed increase in moderately and highly suitable areas in America. The definition of habitat suitability levels could provide insights into making priority areas of pest control. In particular, some South American countries have large rice crops and suitable habitats that may indicate a higher invasion risk.

### 4.4. Integrated Conclusions

By combining evidence from population genetics, demographic history, and ecological niche modeling, our study provides an integrated understanding of the invasion potential of *L. acuta*. The genetic analyses revealed low levels of differentiation and homogeneous genetic components among populations, which can be attributed to strong dispersal ability, frequent gene flow, and wide host use. Demographic inference suggested that populations were not strongly affected by the Last Glacial Maximum, indicating historical stability and persistence of large effective population size. Together, these findings are consistent with the species’ high capacity for dispersal and colonization. Ecological niche modeling further demonstrated that, under current and future climate scenarios, *L. acuta* has potentially suitable habitats far beyond its present distribution, particularly in the Americas and Africa, where rice is extensively cultivated. Taken together, these results highlight the high invasion risk posed by *L. acuta* and emphasize the need for vigilance and preventive strategies in regions with expanding suitable habitats.

## Figures and Tables

**Table 1:** Summary of the locality information used in ddRAD sequencing analyses and population genetic diversity of *Leptocorisa acuta* based on the SNP_80 dataset. π, nucleotide diversity; H_O_, observed heterozygosity; H_E_, expected heterozygosity.

Pop Code	N	Collection Date	Longitude (°E)	Latitude (°N)	SNP_80 Dataset		
					*π*	*H_O_*	*H_E_*
a-FJTX	5	25 May 2016	118.04	24.85	0.0234	0.2430	0.2957
a-GDSW	7	2 Aug 2018	113.57	24.82	0.0208	0.3526	0.3609
a-GDXH	5	17 Jul 2019	111.49	22.32	0.0230	0.2562	0.2915
a-GXNP	5	12 Aug 2020	107.16	22.04	0.0229	0.2535	0.2903
a-GZLM	5	9 Aug 2014	108.06	25.29	0.0223	0.2562	0.2890
a-HNBQ	2	28 Jul 2017	109.27	19.08	—	—	—
a-HNJY	5	1 Aug 2017	108.82	18.70	0.0236	0.2511	0.2962
a-HNTN	4	27 Jul 2017	110.17	19.23	0.0229	0.2960	0.3275
a-LABN	5	11 Aug 2019	104.51	18.24	0.0231	0.2507	0.2821
a-PDC	4	19 Dec 2019	121.35	16.63	0.0224	0.2907	0.3374
a-TLCR	5	10 Jul 2018	101.34	13.03	0.0228	0.2449	0.2849
a-TLMH	4	30 Aug 2018	97.93	19.29	0.0230	0.2907	0.3287
a-TLTM	3	5 Jul 2018	99.14	16.83	0.0241	0.3474	0.4044
a-WMJ	5	13 Apr 2019	100.81	5.01	0.0224	0.2520	0.2876
a-WMPS	5	19 Apr 2019	103.03	3.89	0.0225	0.2508	0.2905
a-YNDF	3	10 Jun 2016	98.56	24.39	0.0222	0.3660	0.4138
a-YNPD	5	4 Aug 2020	103.69	22.97	0.0229	0.2481	0.2897
a-YNXM	6	25 Aug 2010	101.27	21.94	0.0208	0.2165	0.2521

## Data Availability

The raw reads are available at the Sequence Read Archive (SRA) of NCBI under the BioProject accession number PRJNA1026486.

## Supplementary Materials

The following supporting information can be downloaded at https://www.mdpi.com/article/10.3390/insects16090949/s1. Figure S1: The heat map of interpopulation gene flow; Figure S2: The K values by Structure Harvester; Figure S3: Historical demographic changes; Figure S4: The response curves of each environmental variable; Figure S5: The predicted suitable area of *Leptocorisa acuta*; Table S1: Reads number of per sample generated by ddRAD-seq; Table S2: Pairwise genetic differentiation statistics (FST); Table S3: The relative contributions of the environmental variables.
