# Supplementary material for: Assessing the Population Structure and Invasion Risk in Suitable Areas of the Rice Pest Leptocorisa acuta (Hemiptera: Alydidae)
Source: Insects. 2025 Sep 10;16(9):949. doi: 10.3390/insects16090949 (PMC12471163; doi:10.3390/insects16090949)
Supplement: Supplementary file 1 [file insects-16-00949-s001.zip › insects-3833502-supplementary.pdf]

Supplementary Materials for

Assessing the population structure and invasion risk in suitable areas of the rice pest *Leptocorisa acuta* （Hemiptera: Alydidae）

Table of Contents:

Table S1. Reads number of per sample generated by ddRAD-seq

Table S2. Pairwise genetic differentiation statistics (F_{ST})

Table S3. The relative contributions of the environmental variables

Figure S1 The heat map of inter-population gene flow

Figure S2 The K values by Structure Harvester

Figure S3 Historical demographic changes

Figure S4 The response curves of each environmental variable

Figure S5 The predicted suitable area of *Leptocorisa acuta*

Supplementary table S1 Reads number of per sample generated by ddRAD sequencing after removing low-quality reads and demultiplexing.

Sample name	reads number	Sample name	reads number
a-FJTX1	5,726,779	a-LABN1	4,352,599
a-FJTX2	8,035,360	a-LABN2	6,011,181
a-FJTX3	6,965,571	a-LABN3	1,609,774
a-FJTX4	4,391,249	a-LABN4	5,561,958
a-FJTX5	5,032,950	a-LABN5	4,879,448
a-GDSW01	5,003,423	a-TLCR1	6,074,751
a-GDSW02	5,785,564	a-TLCR2	8,658,650
a-GDSW3	9,587,459	a-TLCR3	4,992,593
a-GDSW4	26,773,139	a-TLCR4	2,952,726
a-GDSW5	13,642,991	a-TLCR5	4,618,497
a-GDSW6	31,395,345	a-TLMH1	3,214,728
a-GDSW7	18,873,825	a-TLMH2	8,722,733
a-GDXH1	6,971,723	a-TLMH3	6,624,975
a-GDXH2	10,604,287	a-TLMH4	7,601,765
a-GDXH3	13,374,953	a-TLTM1	10,112,171
a-GDXH4	15,931,073	a-TLTM2	9,216,461
a-GDXH5	12,901,095	a-TLTM3	10,650,270
a-GXNP1	7,198,172	a-WMJ1	8,660,095
a-GXNP2	6,070,475	a-WMJ2	7,615,215
a-GXNP3	11,356,001	a-WMJ3	2,506,632
a-GXNP4	8,671,077	a-WMJ4	2,856,629
a-GXNP5	7,383,782	a-WMJ5	4,194,283
a-GZLM11	3,460,411	a-WMPS1	7,954,315
a-GZLM12	3,160,900	a-WMPS2	7,782,358
a-GZLM13	6,432,677	a-WMPS3	5,772,924
a-GZLM14	7,498,430	a-WMPS4	3,450,860
a-GZLM15	4,489,397	a-WMPS5	5,488,693
a-HNBQ01	8,259,463	a-YNDF1	6,359,805
a-HNBQ02	13,348,290	a-YNDF2	6,282,324
a-HNJY1	11,686,228	a-YNDF3	5,508,886
a-HNJY2	12,151,263	a-YNPD1	3,394,326
a-HNJY3	8,683,485	a-YNPD2	2,915,066
a-HNJY4	14,282,968	a-YNPD3	6,534,743
a-HNJY5	13,904,199	a-YNPD4	4,716,618
a-HNTN1	3,486,335	a-YNPD5	3,819,628
a-HNTN2	5,096,819	a-YNXM01	2,860,394
a-HNTN4	7,331,378	a-YNXM02	1,653,338
a-HNTN5	12,314,369	a-YNXM3	2,057,861
a-PDC1	2,307,697	a-YNXM5	1,754,644
a-PDC2	8,710,202	a-YNXM6	2,486,624
a-PDC3	6,394,847	a-YNXM7	2,581,543
a-PDC4	5,726,023		

Supplementary table S2 Pairwise genetic differentiation statistics (F_{ST}) matrix calculated on ddRAD sequencing data among populations of *L. acuta*.

	a-FJTX	a-GDSW	a-GDXH	a-GXNP	a-GZLM	a-HNBQ	a-HNJY	a-HNTN	a-LABN	a-PDC	a-TLCR	a-TLMH	a-TLTM	a-WMJ	a-WMPS	a-YNDF	a-YNPD	a-YNXM
a-FJTX	0																	
a-GDSW	0.0183*	0																
a-GDXH	0.0188	0.0183*	0															
a-GXNP	0.0190*	0.0186*	0.0190	0														
a-GZLM	0.0193*	0.0189*	0.0190	0.0194	0													
a-HNBQ	0.0261	0.0255*	0.0258	0.0258	0.0261	0												
a-HNJY	0.0211*	0.0209 *	0.0211 *	0.0211*	0.0211 *	0.0262	0											
a-HNTN	0.0211*	0.0206 *	0.0209 *	0.0207	0.0210	0.0265	0.0218	0										
a-LABN	0.0232*	0.0226 *	0.0230 *	0.0227*	0.0229 *	0.0274	0.0220*	0.0234*	0									
a-PDC	0.0320*	0.0319 *	0.0320 *	0.0320*	0.0323 *	0.0372	0.0323*	0.0327*	0.0329*	0								
a-TLCR	0.0224*	0.0222 *	0.0226 *	0.0219*	0.0227 *	0.0268	0.0220*	0.0228*	0.0223*	0.0323*	0							
a-TLMH	0.0244*	0.0239 *	0.0244 *	0.0241*	0.0242 *	0.0286	0.0236*	0.0244*	0.0238*	0.0340*	0.0232*	0						
a-TLTM	0.0249*	0.0243 *	0.0245 *	0.0240*	0.0248 *	0.0290	0.0243	0.0248	0.0245	0.0346*	0.0238	0.0254	0					

a-WMJ	0.0229*	0.0224 *	0.0229 *	0.0225*	0.0228 *	0.0274 *	0.0225*	0.0230*	0.0229*	0.0322*	0.0220*	0.0236*	0.0242*	0				
a-WMPS	0.0229*	0.0227 *	0.0229 *	0.0226*	0.0231 *	0.0276	0.0225*	0.0230*	0.0229	0.0324*	0.0220*	0.0239*	0.0244*	0.0196	0			
a-YNDF	0.0298*	0.0293 *	0.0298 *	0.0291*	0.0293 *	0.0339	0.0285*	0.0297*	0.0285*	0.0387*	0.0287*	0.0257	0.0303	0.0296*	0.0299*	0		
a-YNPD	0.0212*	0.0209 *	0.0212 *	0.0211*	0.0205 *	0.0266	0.0214*	0.0226*	0.0232*	0.0330*	0.0230*	0.0244*	0.0253*	0.0237*	0.0238*	0.0281*	0	
a-YNXM	0.0258*	0.0253 *	0.0260 *	0.0253*	0.0253 *	0.0297 *	0.0247*	0.0260*	0.0248*	0.0353*	0.0247*	0.0232*	0.0267*	0.0258*	0.0261*	0.0227*	0.0244*	0

Note: * denotes significant Fst P values.

Supplementary table S3 The relative contributions of the environmental variables to the Maxent model.

Variable	Percent contribution	Permutation importance
BIO18	42.6	17.9
BIO16	27.7	5
BIO04	9.6	12.8
BIO02	5.3	8.4
BIO01	3.6	19.6
BIO13	2.1	0.4
BIO03	1.6	5.4
BIO06	1.2	0.7
BIO17	1.2	0.8
BIO15	1.1	7.2
BIO10	1.1	7.2
BIO12	0.7	0.7
BIO14	0.7	2.6
BIO08	0.5	2.4
BIO19	0.4	0.5
BIO05	0.2	4.2
BIO07	0.2	0.2
BIO11	0.1	3.6
BIO09	0	0.6

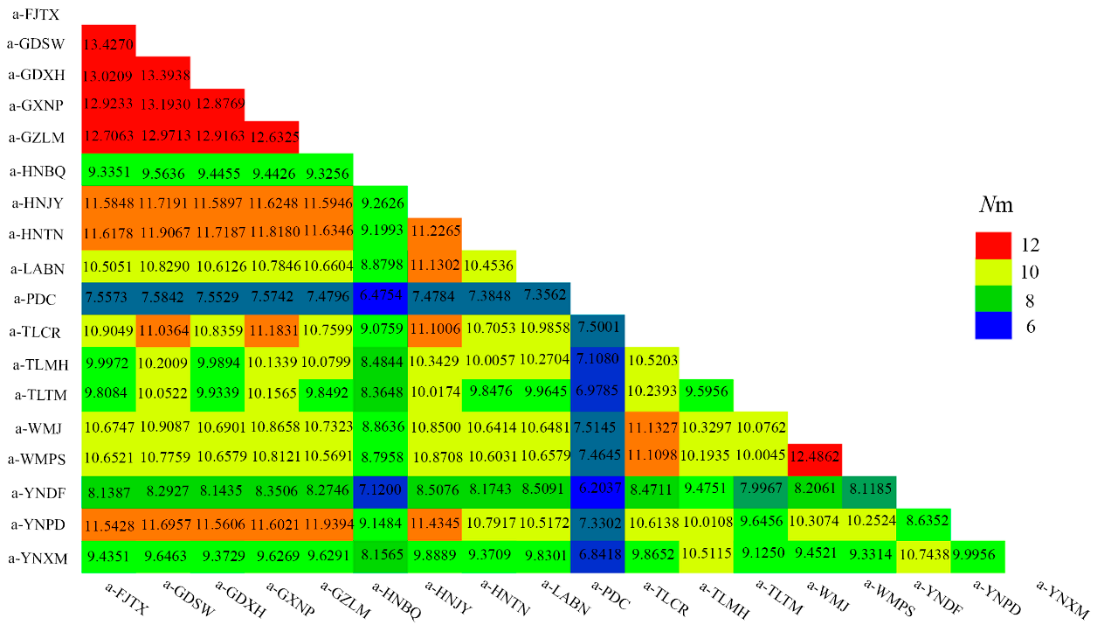

Figure S1 The heat map of inter-population gene flow estimated by Nm.

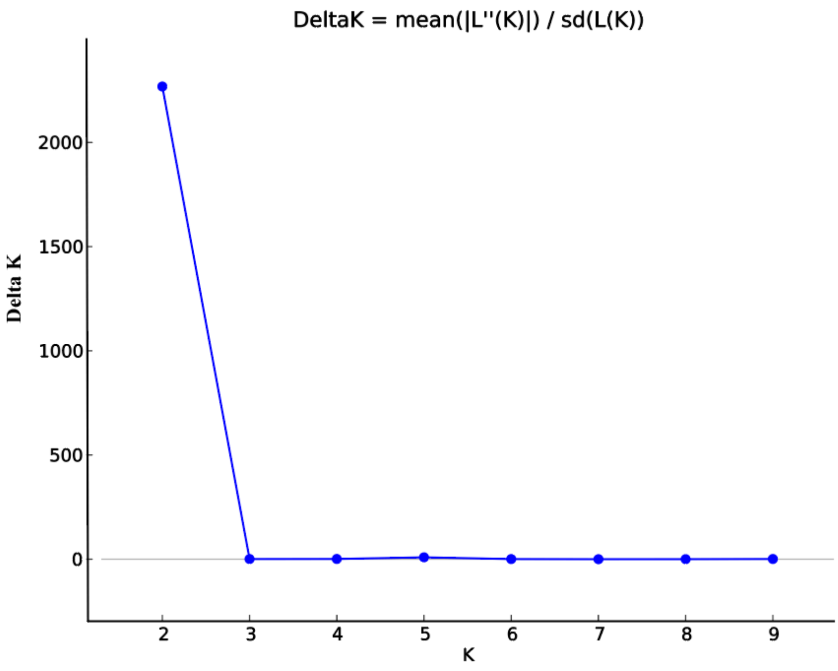

Figure S2 The K values by Structure Harvester (K=1~10) showed the best K=2.

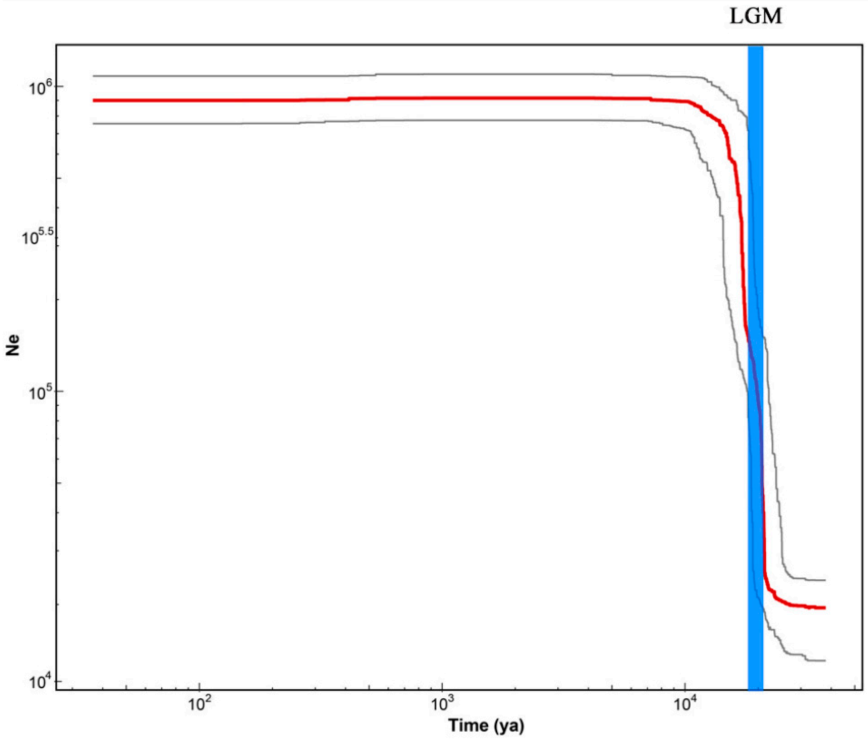

Figure S3 Historical demographic changes based on Stairway plot (thick red lines represent); dark gray lines represent 95% pseudo-confidence intervals. The X-axis represents time (years ago), and the Y-axis represents the estimated scaled effective population size. LGM-last glacial maximum

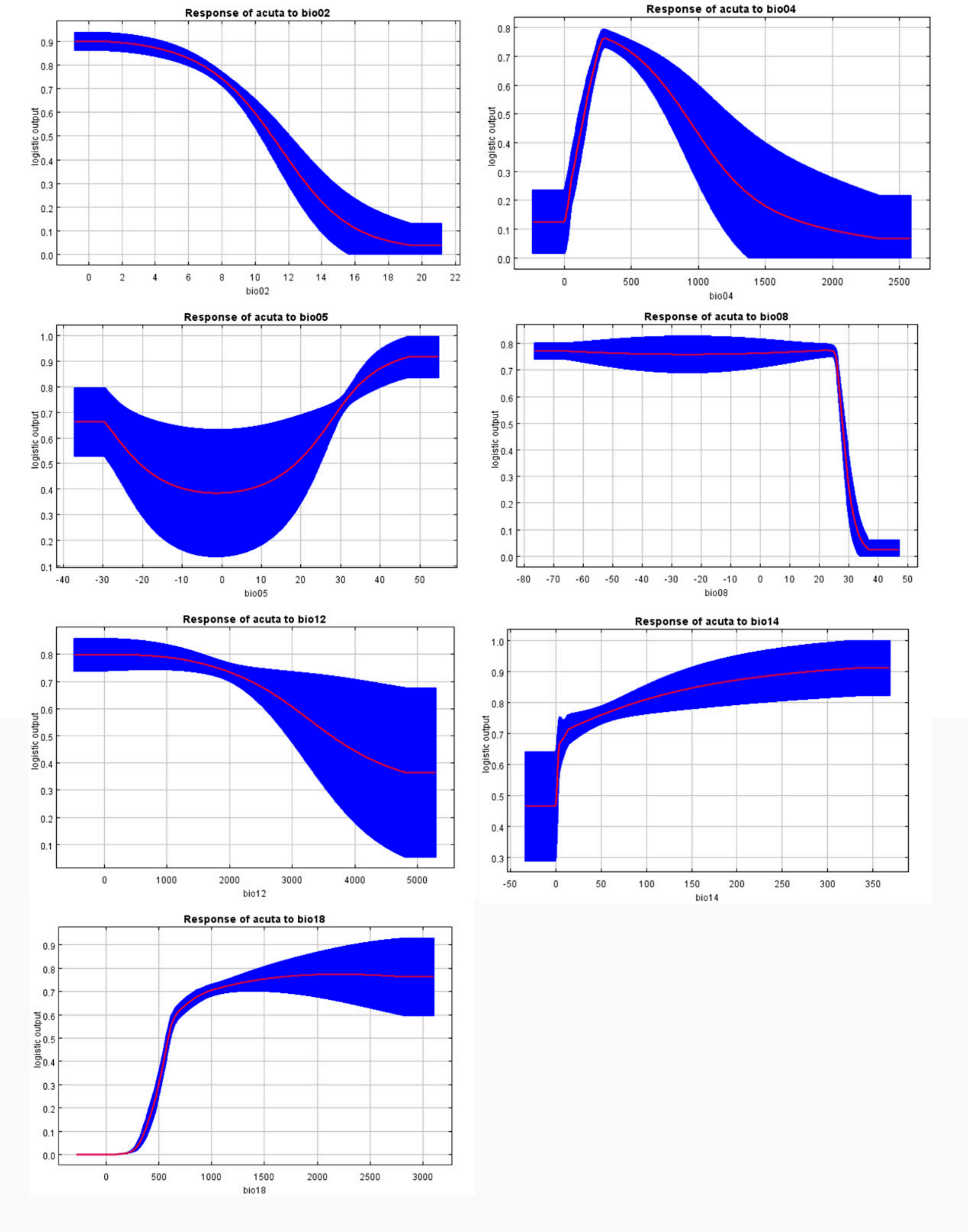

Figure S4 The response curves show how each environmental variable affects the Maxent prediction. The curves show the mean response of the Maxent runs (red) and the standard deviation (blue).

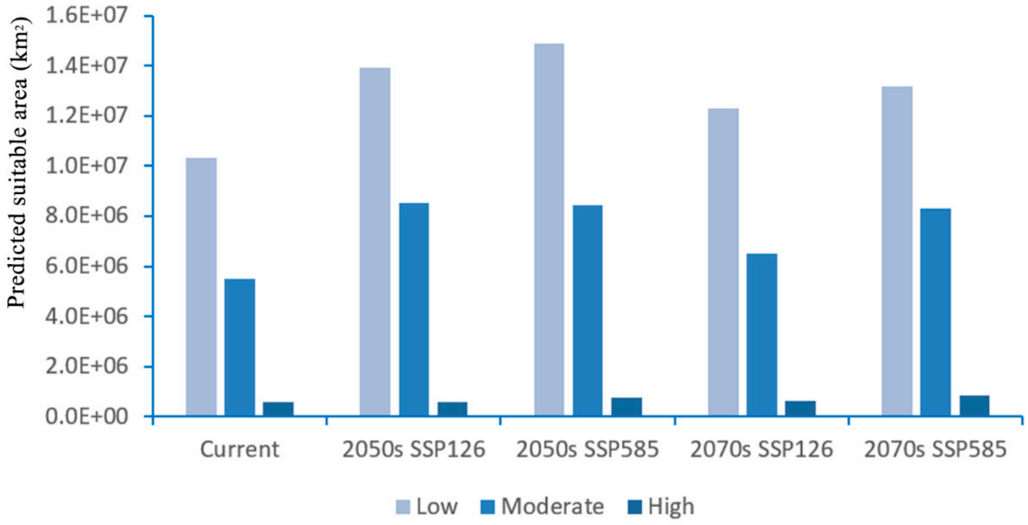

Figure S5 The area of suitable habitats predicted under current and future climatic conditions.
